# PET imaging of microglia using PBR28suv determines therapeutic efficacy of autologous bone marrow mononuclear cells therapy in traumatic brain injury

**DOI:** 10.1038/s41598-023-43245-0

**Published:** 2023-09-26

**Authors:** Supinder S. Bedi, Michael C. Scott, Max A. Skibber, Akshita Kumar, Henry W. Caplan, Hasen Xue, David Sequeira, Alison L. Speer, Fanni Cardenas, Franciska Gudenkauf, Karen Uray, Amit K. Srivastava, Alan R. Prossin, Charles S. Cox

**Affiliations:** 1 Department of Pediatric Surgery, University of Texas Medical School at Houston, 6431 Fannin Street, MSB 5.230, Houston, TX 77030 USA; 2 Division of Hematology, Department of Medicine, Cardeza Foundation for Hematologic Research, Sydney Kimmel Medical College, Thomas Jefferson University, Philadelphia, USA; 3 Department of Psychiatry and Behavioral Sciences, University of Texas Medical School at Houston, Houston, TX USA

**Keywords:** Glial biology, Neuroimmunology, Regeneration and repair in the nervous system

## Abstract

Traumatic brain injury (TBI) results in activated microglia. Activated microglia can be measured in vivo by using positron emission topography (PET) ligand peripheral benzodiazepine receptor standardized uptake values (PBR28suv). Cell based therapies have utilized autologous bone marrow mononuclear cells (BMMNCs) to attenuate activated microglia after TBI. This study aims to utilize in vivo PBR28suv to assess the efficacy of BMMNCs therapy after TBI. Seventy-two hours after CCI injury, BMMNCs were harvested from the tibia and injected via tail-vein at 74 h after injury at a concentration of 2 million cells per kilogram of body weight. There were three groups of rats: Sham, CCI-alone and CCI-BMMNCs (AUTO). One hundred twenty days after injury, rodents were imaged with PBR28 and their cognitive behavior assessed utilizing the Morris Water Maze. Subsequent ex vivo analysis included brain volume and immunohistochemistry. BMMNCs therapy attenuated PBR28suv in comparison to CCI alone and it improved spatial learning as measured by the Morris Water Maze. Ex vivo analysis demonstrated preservation of brain volume, a decrease in amoeboid-shaped microglia in the dentate gyrus and an increase in the ratio of ramified to amoeboid microglia in the thalamus. PBR28suv is a viable option to measure efficacy of BMMNCs therapy after TBI.

## Introduction

Traumatic Brain Injury (TBI) causes a prolonged secondary neuroinflammatory response within the central nervous system (CNS) that can amplify neurological deficits, both motor and cognitive, beyond that caused by the primary injury^1,2^. Central to the secondary inflammatory response after TBI are the activation of resident microglia^3^. Microglia promote learning dependent synapse formation, axonal regeneration, and removal of defunct axon terminals^4,5^. Under homeostasis, microglia are highly mobile and provide continuous surveillance of their cellular milieu^3,6^. These “resting” or ramified microglia possess a distinct morphology—small, relatively stable rod-shaped somata with thin ramified withdrawing processes^7,8^. After TBI, due to the disruption of the blood brain barrier (BBB) there is an influx of macrophages and microglia are activated. The infiltrating peripheral macrophages are also responsible, in part, in activating resident microglia^9^. Once activated, microglia are critically involved in helping reseal the ruptured BBB^10^. Activated microglia retract their processes and typically adopt an amoeboid morphology^6,11,12^. Morphological alterations may also be accompanied by alterations in mitochondria^13^.

We have previously demonstrated that there is a significant increase in amoeboid microglia/macrophages after TBI in the dentate gyrus and thalamus^8,14–16^. Additionally, in patients, the corpus callosum is surrounded by activated microglia^17^. Once activated, microglia can polarize towards a pro-inflammatory phenotype which can result in chronic neuroinflammation, oxidative stress and neuronal dysfunction. On the other hand, microglia can also adopt anti-inflammatory functions, and can result in resolution of inflammation, clearance of debris and neural repair. The pro- or anti-inflammatory phenotype is dependent upon the local environment and timing after injury^17^.

Another protein that increases in expression after injury is the peripheral benzodiazepine receptor (PBR), expressed by mitochondria in microglia, reactive astrocytes, blood immune cells, endothelial and smooth muscle cells in the vasculature^13,18–20^. PBR is localized on the outer mitochondrial membranes of astrocytes, microglia and macrophages^21^. It is part of a hetero-oligomeric complex comprised on the voltage-dependent anion channel and an adenine nucleotide carrier forming the mitochondrial permeability transition pore^22,23^.

PBR can be detected in rodents and patients using positron emission topography (PET) ligands. Evidence from human TBI patients using ligand PK11195 suggests that amoeboid microglia/macrophages and/or reactive astrocytes remain present up to 17 years after injury^1^. Second generation ligands with higher affinity and specificity for PBR include PBR28^24^. However, binding of PBR28 is determined by genetic variation in the PBR gene in humans but not in rodents^25^. We have recently demonstrated an increase in PBR28suv after CCI (a rodent model of TBI) using [^11^C]PBR-28^26^. All these data suggest there is prolonged inflammation as measured by activated microglia/macrophages and that [^11^C]PBR-28 is a viable marker to study activated microglial cells in patients as well as in rodents.

Recent advances in cell therapy have utilized autologous bone marrow-derived mononuclear cells (BMMNCs) for therapeutic outcome for central nervous system insults such as stroke and TBI in humans and in rodent models^27,28^. BMMNCs (2 million cells/kg) treatment at 74 h post TBI (rodent model) protects the brain by preserving the blood brain barrier (BBB) and attenuating the amoeboid shaped microglia in the ipsilateral hippocampus via apoptosis, resulting in improved cognitive behavior^15^. It would be useful to investigate a biomarker that can be used in vivo to quantify changes due to cellular therapy. The aim of this is study is to determine if PBR28suv is an appropriate measure of efficacy of BMMNCs therapy after TBI.

## Methods

**Figure 1:**
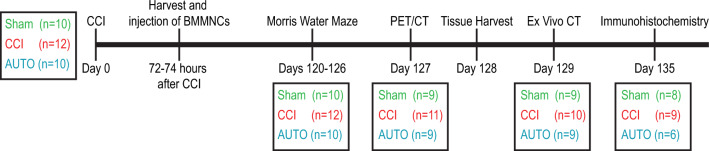
Time line of experimental paradigm. Three groups of animals: Sham, CCI and AUTO (CCI + BMMNCs). The different outcomes are from the same animals that we started the experimental set up (Sham: n = 10, CCI: n = 12 and AUTO: n = 10). Injury was done on Day 0, followed by harvest and injection of BMMNCs (72–74 h after CCI), Morris water maze [Days 120–127 (Sham: n = 10, CCI: n = 12 and AUTO: n = 10)], PET/CT [Day 128 (Sham: n = 9, CCI: n = 11 and AUTO: n = 9)], tissue harvest (Day 129), ex vivo CT [Brain volumetrics, Day 130 (Sham: n = 9, CCI: n = 10 and AUTO: n = 9)] and immunohistochemistry (Day 135) (Sham: n = 6, CCI: n = 9 and AUTO: n = 6)].

Adult male Sprague Dawley rats aged six to eight weeks (Harlan/Envigo, Indianapolis, IN, USA) were housed on a twelve-hour light/dark cycle with ad libitum access to food and water. We used male rats because of our previous experiments, and the number of animals used was also based on our previous publications^8,15,29^. There were three groups of animals: Sham, CCI and AUTO (CCI + BMMNCs therapy) and the timeline of the experiments is in Fig. 1 Total animals used were (Sham: n = 10, CCI: n = 12 and AUTO: n = 10). Animals that were used for behavior (Sham: n = 10, CCI: n = 12 and AUTO: n = 10) were the same animals that were PET-imaged (Sham: n = 9, CCI: n = 11 and AUTO: n = 9) followed by brain-volume metrics (Sham: n = 9, CCI: n = 10 and AUTO: n = 9) and immunohistochemistry (Sham: n = 6, CCI: n = 9 and AUTO: n = 6). The changes in the PET-imaged animals and brain-volume metrics are due to elimination of outliers. The changes in the sample size for immunohistochemistry is due to damaged brains during vibratome slicing. All protocols involving the use of animals were compliant with the National Institutes of Health Guide for the Care and Use of Laboratory Animals and were approved by the Institutional Animal Care and Use Committee (HSC-AWC-15-0003).

### Controlled cortical impact (CCI) model of traumatic brain injury

Prior to creation of the TBI, each animal went through a pre-operative checklist to maximize survival following the CCI. Each animal was initially anesthetized with 4% isoflurane with a 1:1 N_2_O/O_2_ mixture in a vented anesthesia chamber. When the animal failed to respond to foot and tail pinch, they were removed from the anesthesia chamber and continually anesthetized with a 2–2.5% isoflurane mixture via facemask. Aseptic surgical technique was used for the surgical procedure. Body temperature was monitored by a rectal thermometer and regulated by the use of a heating pad throughout the operation. Prior to any incision, the subcutaneous tissue was infiltrated with < 0.1 mL/kg of 0.25% Bupivacaine.

The CCI began with a midline scalp incision and the right-sided soft tissue was reflected laterally to expose the skull. Unilateral craniectomy was made midway between the bregma and the lambda (3 mm right of midline) with the medial edges adjacent to the midline suture. A single impact was performed using a sterile impactor tip. The scalp was then stapled closed with sterile wound clips.

In order to assess the spatial and temporal microglia response to injury, the rats were randomly selected to undergo either a sham injury or a right sided CCI (Device: Impact One Stereotaxic Impactor, Leica Microsystems, Buffalo Grove, IL). CCI severity is based on injury depth, and was measured independently by a device attached to the impactor tip (6 mm). The severe injury consisted of a 2.7 mm impact depth at a velocity of 5.6 m/s, dwell time 200 ms. The sham procedure entailed a midline incision and reflection of the soft tissue laterally to expose the skull. A craniectomy was not performed in this animal group. A traditional sham operation that incorporates craniectomy results in profound inflammatory and anatomical damage that may severely confound results of a TBI animal model, therefore it has become standard to not perform a craniectomy for sham. This methodology has remained standard in our lab, which focuses primarily on rodent TBI models^30^.

### Bone marrow harvest

Seventy-two hours after CCI injury, the treatment group of rats were anesthetized with 4% isoflurane and O_2_ in the supine position. The anterior surface of the bilateral lower legs were scrubbed with betadine. A 2.5 cm incision was made on the anterior surface of the lower leg. Next the musculature was carefully dissected medially to expose the anterior surface of the tibia. A small burr hole was drilled into the anterior surface of the tibia approximately 1 cm below the tibial plateau. Next, using a 10 mL syringe with a 23 gauge needle, the bone marrow was aspirated from the bilateral tibias. After harvest, the bone marrow was placed in heparanized tubes and placed on ice until processing. The skin incisions were then closed with 3-0 nylon suture in a running fashion. In order to prevent any additional painful stimuli (drilling a hole in the tibia) and to blind the operator during cognitive testing, Sham and CCI control groups only received skin incisions with 3-0 nylon suture in a running fashion on the bilateral lower legs. Furthermore, we did this to mimic the clinical trial shams in which IRBs do not allow us to drill into the bone for the placebo control, rather just a skin nick for blinding, and as close as reasonable to the harvest procedure. The body temperature was maintained at 37 °C by the use of a heating pad. Previously obtained serial arterial PaO2 and PaCO2 measurements have shown that animals do not become hypoxic or hypercarbic during this procedure^31^. For all experiments, BMMNC was harvested at 72 h and injected 74 h after injury.

### Bone marrow mononuclear cell isolation

After harvest, the bone marrow was filtered through a 100 µm sized filter to remove any residual bone fragments or remaining adipose tissue. Next, the bone marrow samples were diluted 2:1 with Hank's balanced salt solution (HBSS). The diluted solution was then layered over an equal volume of Ficoll-Hypaque (GE Healthcare, Piscataway, NJ) solution in a 50 mL conical tube. The samples were placed in the centrifuge at 1000×*g* for 30 min. The BMMNCs were contained in the upper fluid interface and were carefully aspirated out of the conical tube and transferred to a separate 15 mL conical tube. The samples were then washed with 4% fetal bovine serum in phosphate buffered saline (PBS) and placed in the centrifuge at 1000×*g* for 3 min. This step was repeated for a total of two washes. The BMMNCs were then suspended in PBS. Cells were counted and checked for viability via Trypan blue exclusion and suspended at a concentration of 2 × 10^6^ cells per kilogram in 1 mL. Immediately prior to intravenous injection, the cells were vortexed gently 8–10 times to ensure a homogeneous mixture of cells. All three groups (Sham, CCI and AUTO) were anesthetized and placed in the supine position. A tail vein injection was completed followed by injection of the cell suspension for the treatment group (AUTO) and PBS vehicle alone for the Sham and CCI injury alone groups.

### Latency to platform

All behavior testing were done under blinded conditions, with unblinding after all the behavior testing was done. Cognitive function was tested using the Morris Water Maze (MWM) 120 days after injury to assess spatial memory and spatial learning. Animals were tested using four trials per day, over 6 consecutive days. Each trial consisted of placing the animal in one of four starting locations (south, east, west, and north) chosen at random. The animal was gently placed in the tank facing the wall and allowed to search for the platform (located in north quadrant) for up to 60 s. If the animal failed to find the platform, it was placed upon the platform and allowed to remain for 30 s. Animal movement within the maze was monitored by a video camera linked to tracking software (Ethovision 3.1). Latency to platform was measured.

### Probe

To test memory retention, probe trials were administered 24 h (day 126) after completion of the platform testing. Probe testing (60 s) involved removal of the platform and, using the tracking software, monitoring the animal movement. Calculations were then completed to determine the time spent in the area three times the size of the platform (platform proximity duration). In addition, we also measured the number of times the animal crossed the location of the hidden platform.

### Synthesis of [^11^C]PBR-28

The radioligand [^11^C]PBR-28 was purchased and synthesized from the University of Texas MD Anderson Cancer Center Cyclotron Radiochemistry Facility. In brief, [^11^C]CO_2_ is produced in the cyclotron target by the N_2_ + 1% O_2_ mixture via the nuclear reaction ^14^N_(p,α)_^11^C. After irradiation, the [^11^C]CO_2_ from the target is transferred to a processing module where it is first trapped and then converted to [^11^C]methane. Subsequently, the [^11^C]methane is reacted to yield [^11^C]methyl iodide. The [^11^C]CH_3_I is then passed through another column containing silver triflate at elevated temperatures, which in turn produces [^11^C]methyl triflate. The [^11^C]methyl triflate is then transferred to a second automated synthesis module where it reacts with the precursor. After the reaction, the solution is injected into a Prep HPLC column where the final product is purified. From this second module, the radiopharmaceutical is passed through a sterilizing filter into a sterile final product vial. A sample from the final product vial is removed and analyzed to ensure the product meets release criteria. Finally, the vial is moved to the radio-pharmacy for individual dose dispensing.

### PET imaging

Imaging was performed at the MD Anderson Cancer Center for Advanced Biomedical Imaging (CABI)/Small Animal Imaging Facility (SAIF) 127 days after CCI. Rats were anesthetized using 2% isoflurane via facemask. At the start of the PET scan, the rats were injected with 300 uCi of [^11^C]PBR-28 in 200-μL of sterile saline via a tail vein catheter. Each rat was imaged with a sixty-minute PET scan and a five-minute CT scan (400 μA, 45 kV, 120 projections) on a Bruker Albira PET/SPECT/CT scanner (Bruker Biospin Corp., Billerica, MA, USA).

### Imaging data analysis

The PET data was reconstructed into the following bins: 15 × 20 s, 5 × 60 s, and 4 × 300 s using a maximum likelihood expectation maximization (MLEM) method. Analysis was performed with P_mod_ (PMOD Technologies Ltd., Zürich, Switzerland). Using the skull outline from the CT image, two regions were drawn to cover the two cerebral hemispheres of the brain. The time-activity curve developed was PBR uptake in each region over the period of the PET scan was expressed as Standard Uptake Values [SUV (g/ml)]. This allows for body mass and minor variations in injected dose. Scatter, randoms, decay, and attenuation corrections were applied.

The SUV has become widely used in PET imaging analysis, making it an excellent tool for comparing radioligand uptake amongst our three different TBI injury severity groups. The SUV is considered a semi-quantitative analysis defined as the ratio of tracer activity in the tissue of interest divided by normalizing tracer activity (background activity, organ activity, etc.^32^. The regions of interest (ROIs) included the ipsilateral-injured cerebral hemisphere. Soft tissue uptake of the radioligand [^11^C]PBR-28 was excluded from analysis, so only intracranial microglia activation was evaluated. The SUV was determined by the ratio of mean intracranial SUV to that of muscle. Contralateral brain hemisphere normalization was not possible, as contrecoup injuries would artificially lower mean SUVs in the severe injury group.

### Tissue harvest

On day 128 after injury, the animals were euthanized. The animals first underwent bilateral thoracotomies under isoflurane anesthesia. Then, using a right ventricle puncture technique, the animals were simultaneously exsanguinated and perfused with 20 mL of cold phosphate buffered saline (PBS). Following the PBS infusion, 20 mL of freshly prepared, cold 4% paraformaldehyde (PFA) was instilled to reduce non-specific binding and AUTO-fluorescence. The brains were carefully removed and post-fixed with 4% PFA for 24 h while stored at 4 °C.

### Brain volume

On day 129, ex vivo CT imaging was performed with a high-resolution GE Ultra flat panel CT scanner (General Electric, Milwaukee, WI) with the following acquisition settings: 80 kVp, 22 mA with 16 s rotation/ exposure. Simple back projections were obtained from the 0.154 µm image reconstruction and exported as DICOM images. Image analysis was performed using the OsiriX software. In order to provide a qualitative and quantitative analysis of the injury and the whole brain, an ROI volume of the injury or the brain hemispheres was created in a 2D reconstruction. A series of ROIs of the injury were first manually created in consecutive CT slices. Then, all the ROIs in the series were saved, the missing ROIs were computed, and the volume of the injury or the hemispheres was calculated, exported and saved as a DICOM file. The process is called segmentation and it helps identify and locate lesions or injuries. It also provides precise measurements of the injury’s volume. Additionally, the corresponding three-dimensional volume rendering models were acquired for all the brain samples. The measurements were done by blinded trained personnel.

### Immunohistochemistry

On day 130 after injury, the brains were transferred to a 30% sucrose solution, where they were maintained at 4 °C for at least 72 h and allowed to sink. Brains were then put in a 3% melted agar mold and sectioned into 30-μm-thick slices using a vibrating-blade microtome (Leica Microsystems, Bannockburn, IL). Two histological sections per animal approximately from mid-injury (interaural 5.70 mm, bregma − 3.30 mm) were examined. Brain sections were stained for microglia using a free-floating protocol. The protocol was carried out over several days. On day one, the brain slices were transferred to twelve well plates and washed twice in PBS with 0.01% Triton X-100 (PBST; T-8787; Sigma-Aldrich, St. Louis, MO) for 1 min. Next, the slices were incubated for 20–30 min in PBS with 0.02% Triton X-100 for permeabilization. The slices were then blocked for one hour in blocking buffer consisting of 3% goat serum (no. 005-000-121; Jackson Immunoresearch Laboratories, West Grove, PA) in PBST. The last step of day one involved incubating the brain slices with the primary antibody IBA-1, which is used to identify microglia/macrophage morphology (rabbit polyclonal primary antibody, 1:500; Wako Chemicals USA, Cat# 019-19741, RRID: AB_2313566). The primary antibody was prepared in PBTB [PBST, 2% bovine serum albumin (A9647; Sigma-Aldrich) and 1% goat serum], and sections were incubated overnight at 4 °C. The following day, sections were washed with PBST and incubated with a goat anti-rabbit IgG secondary antibody (1:500; red/568; Molecular Probes (Invitrogen) Cat# A11011, RRID: AB143157) in PBTB for 2 h at room temperature. The sections were again rinsed four times with PBST, mounted on slides, and cover-slipped with Fluoromount-G (SouthernBiotech) for analysis.

### Microglia morphology quantification

Ex vivo analysis in AUTO, CCI and Sham were done by quantifying microglia morphology (ipsilateral) with photomicrographs of the injury dentate gyrus (hippocampus) and thalamus (5 photomicrographs/slice X 2 slices/animal). Photomicrographs were taken at 20X magnification using a Leica fluorescent microscope Dm4000B LED (https://www.leica-microsystems.com/products/light-microscopes/p/leica-dm4000-b-led) and Leica Application Suite V4.12 (https://www.leica-microsystems.com/products/microscope-software/p/leica-application-suite). Two slices per animal was examined and the sections of interest were approximately mid-injury (small cavity or bruise left by the impactor tip). IBA-1 labeled cells were further quantified based on the following microglia morphologies: ramified or amoeboid as previously described^14^.

### Statistical analysis

All data are expressed as means ± standard deviation. Statistical analysis was performed with Prism software (version 7.0b; GraphPad Software Inc., La Jolla, CA). In vivo PBR28suv were analyzed with Two-way analysis of variance (ANOVA) with multiple comparisons utilizing Uncorrected Fisher’s LSD. Morris Water Maze were analyzed with Two-way analysis of variance (ANOVA) with multiple comparisons utilizing Uncorrected Fisher’s LSD. Ex vivo immunohistochemistry were analyzed with Two-way analysis of variance (ANOVA) with multiple comparisons utilizing Uncorrected Fisher’s LSD. Brain Volume were compared using unpaired non-parametric Mann–Whitney test. Outliers were excluded using ROUT with Q = 1%. Statistical significance is indicated with * for p < 0.05, ** indicates statistical significance for p < 0.01, *** indicates statistical significance p < 0.001, and **** indicates statistical significance p < 0.0001.

### Ethical approval

All methods are in accordance with ARRIVE guidelines. All protocols involving the use of animals were in compliance with the National Institutes of Health Guide for the Care and Use of Laboratory Animals and were approved by the University of Texas Health Science Center Institutional Animal Care and Use Committee (AWC-140122).

## Results

### BMMNCs therapy attenuated PBR28suv in comparison to CCI alone

**Figure 2:**
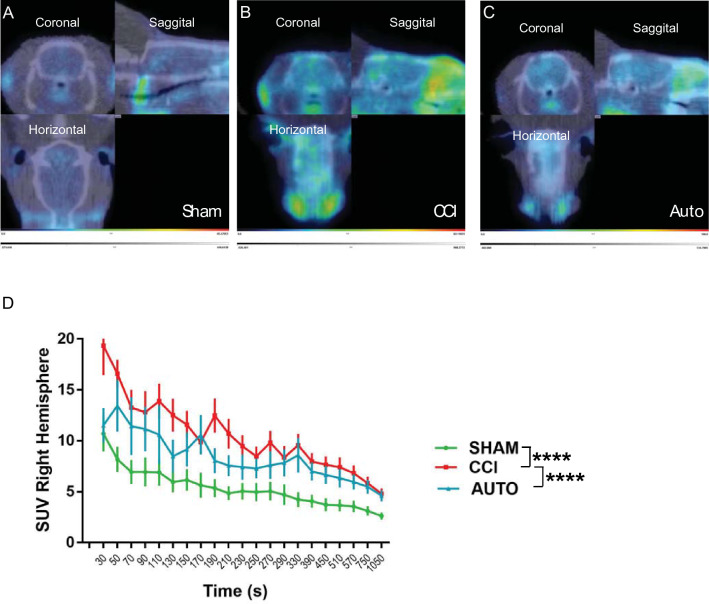
BMMNCs therapy decreases PBR28suv after TBI 128 days after injury. Representative PET images of (**A**) Sham, (**B**) CCI and (**C**) AUTO. The regions of interest (ROIs) included the ipsilateral-injured cerebral hemisphere. SUV was determined by the ratio of mean intracranial SUV to that of muscle. (**D**) There were significant differences due to time (P < 0.0001) and treatment (P < 0.0001) of PBR28suv from 30 to 1050 s.

The time–activity curves of the ipsilateral hemispheres demonstrates that BMMNCs therapy after TBI attenuates PBR28suv in comparison to CCI alone (Fig. 2A–D). When comparing the PBR28suv (ipsilateral) over time, there were significant increases in PBR28suv in time (P < 0.0001) and treatment (P < 0.0001, Fig. 2). Subsequent multiple comparisons using Uncorrected Fishers’ LSD test demonstrated a significant decrease in PBR28suv between AUTO (n = 9) and CCI (n = 11, P < 0.0001, Fig. 2). Additionally, there was a significant decrease in PBR28suv between Sham (n = 9) and CCI (P < 0.0001, Fig. 2).

### BMMNCs therapy improved spatial learning as measured by the Morris water maze

**Figure 3:**
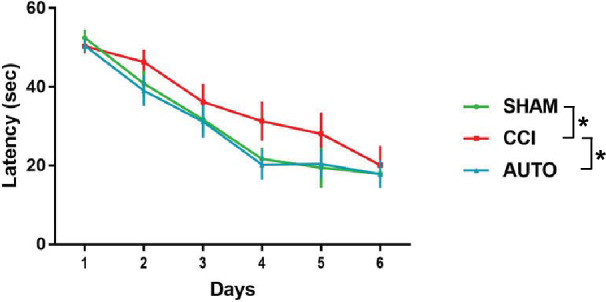
BMMNCs therapy administered seventy-two hours after traumatic brain injury improved spatial learning and memory as measured by the attenuation in latency to find the hidden platform 120 days after injury. Six-day spatial training paradigm in Morris water maze. There were significant differences due to time (P < 0.0001) and treatment (P < 0.05). There are significant difference between CCI and AUTO (P < 0.01) and CCI and Sham (P < 0.04).

In order to measure spatial learning, we used the Morris Water Maze (MWM) spatial learning paradigm. Spatial learning was conducted on days 120–125 after TBI (Fig. 3A) followed by a probe trial on day 126 (see “Methods”). There were significant effects of treatment (P < 0.03) and time (P < 0.0001). Subsequent multiple comparisons using Uncorrected Fishers’ LSD test demonstrated a significant decrease in latency to platform between AUTO (n = 10) and CC I (n = 12, P < 0.05). Additionally, there was a significant decrease in latency to platform between Sham (n = 10) and CCI (P < 0.05).

### BMMNCs therapy does not improve probe testing

Although there was increase in the number of times, the platform was crossed by AUTO (n = 10, 3.4 ± 0.5) in comparison to CCI [(n = 12, 2.4 ± 0.6, p > 0.05)]. There were no differences between CCI and Sham (n = 10, 2.4 ± 0.6). Additionally, there were no differences in the time (seconds) spent in the area 3 times the size of the platform [Sham (n = 10, 5.8 ± 1.0), CCI (n = 12, 4.7 ± 0.5) and AUTO (n = 10, 5.5 ± 1.1), p > 0.05)].

### BMMNCs therapy prevents brain volume loss

**Figure 4:**
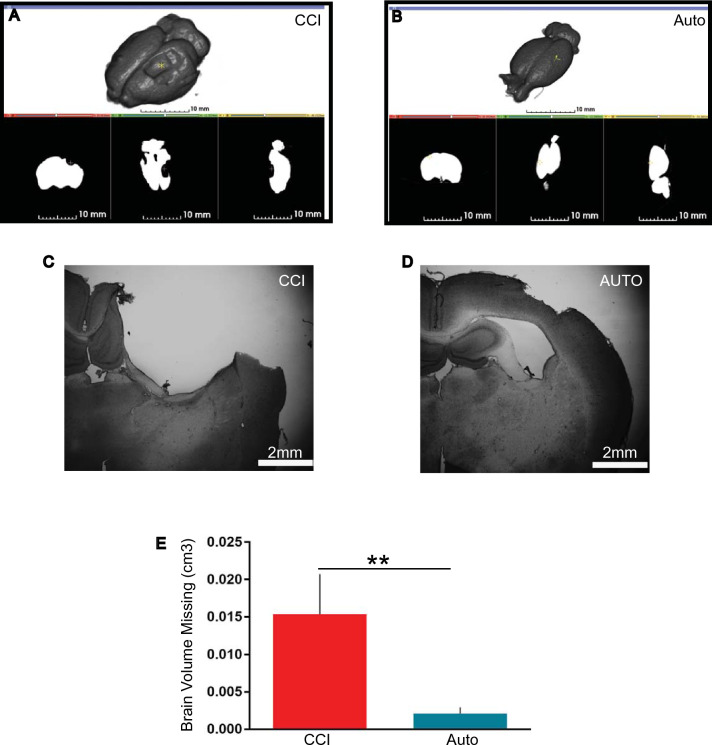
BMMNCs therapy prevent brain volume loss. (**A**) Ex vivo brain images of CCI and (**B**) AUTO 128 days after injury were compiled using the 3D Slicer open software platform. (**C**) Examples of Nissl stained brains of CCI and (**D**) AUTO. (**E**) Graph representing volume loss (cm^3^). There was a significant decrease in volume loss in BMMNCs treated group in comparison to CCI (P < 0.01).

Ex vivo analysis demonstrated BMMNCs therapy after TBI results in preservation of brain volume as measured by X-ray-based Computed Tomography (CT) imaging followed by “Threshold Effect” (see “Methods”). Specifically, there was a decrease in the brain volume (mm^3^) in CCI (n = 10, 0.014 ± 0.01) when compared to BMMNCs treated brains (Fig. 4, p < 0.01, n = 9, 0.006 ± 0.003). There was no volume loss in the Sham brains.

### BMMNCs therapy reduces amoeboid microglia in the dentate gyrus

**Figure 5:**
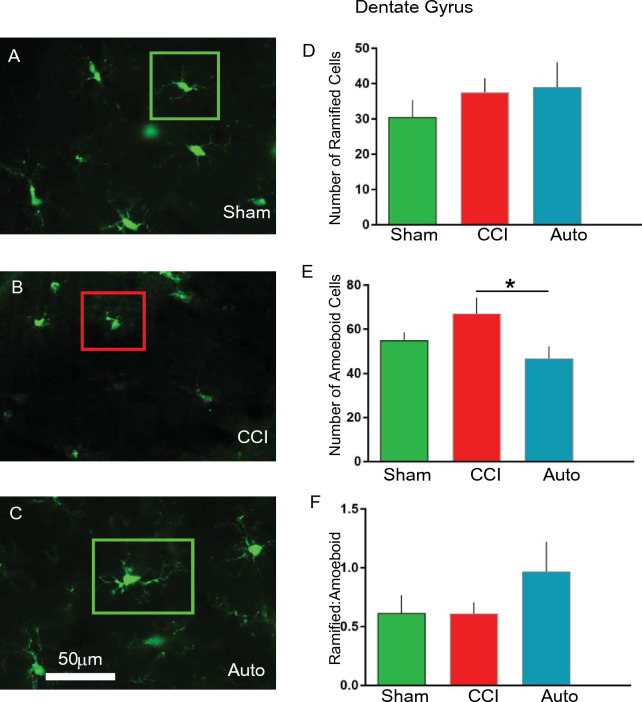
BMMNCs therapy attenuates amoeboid-shaped microglia in the dentate gyrus after TBI. Representative photomicrographs of (**A**) Sham, (**B**) CCI and (**C**) AUTO. Green squares indicate ramified microglia and red squares indicate amoeboid microglia. (**D**) Graph comparing number of ramified cells between Sham, CCI and AUTO. (**E**) Graph comparing number of amoeboid cells between Sham, CCI and AUTO. There is a significant decrease in the number of amoeboid cells when comparing CCI vs AUTO (P < 0.05). (**F**) Graph comparing Ramified:Amoeboid cells between Sham, CCI and AUTO.

There was a significant decrease (P < 0.03) in amoeboid microglia as measured by IBA1 immunohistochemistry due to BMMNCs therapy (AUTO: n = 6, 47 + 5) in comparison to CCI (n = 9, 67 + 7), (Fig. 5E). There was an increase in amoeboid microglia in CCI (n = 9, 67 + 7) in comparison to sham (n = 8, 55 + 4), however it was not significant. Neither CCI (n = 9, 38 + 4) nor BMMNCs therapy (n = 6, 37 + 7) affected ramified microglia in the dentate gyrus (Fig. 5D). The number of sham microglia (n = 8, 30 + 5) in the dentate gyrus were also similar to the other groups and not significant. We also compared the ratio of ramified vs amoeboid microglia. While there was an increase in AUTO (n = 6, 0.97 + 0.2) vs CCI (n = 9, 0.6 + 0.1), it was not significant. Surprisingly, there was no difference between CCI (n = 9, 0.6 + 0.1) and sham (n = 8, 0.6 + 0.1, Fig. 5F).

### BMMNCs therapy increases ramified vs amoeboid microglia in the thalamus

**Figure 6:**
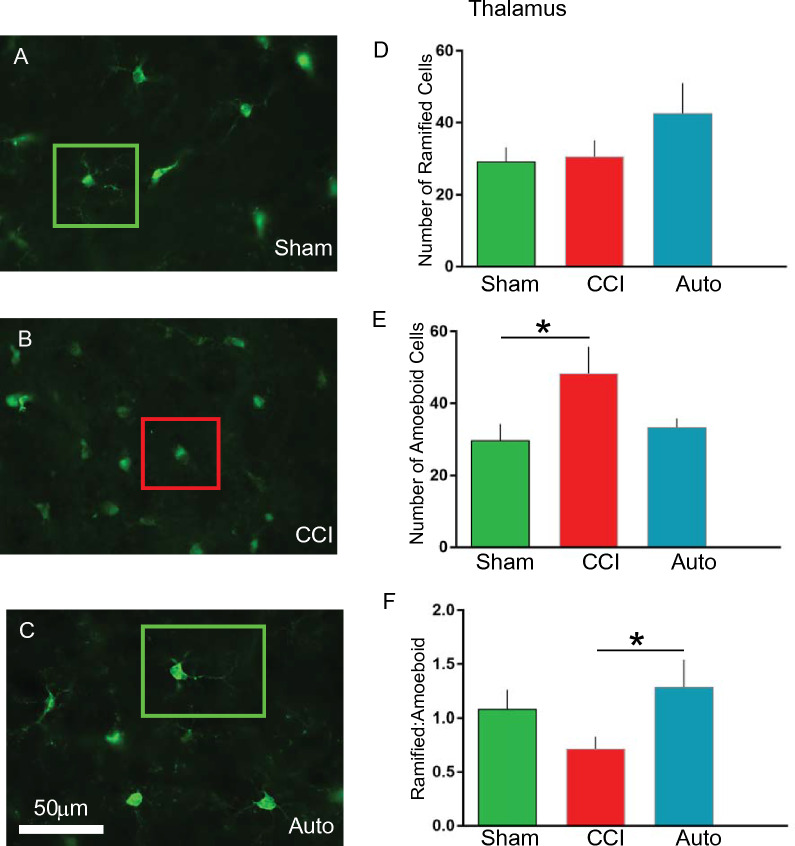
BMMNCs therapy increases the Ramified:Amoeboid in the thalamus after TBI. Representative photomicrographs of (**A**) Sham, (**B**) CCI and (**C**) AUTO. Green squares indicate ramified microglia and red squares indicate amoeboid microglia. (**D**) Graph comparing number of ramified cells between Sham, CCI and AUTO. (**E**) Graph comparing number of amoeboid cells between Sham, CCI and AUTO. There is a significant decrease in the number of amoeboid cells when comparing CCI vs Sham (P < 0.05). (**F**) Graph comparing Ramified:Amoeboid cells between Sham, CCI and AUTO. There is a significant increase in AUTO (Ramified:Amoeboid) when compared to CCI (P < 0.05).

There was a significant increase (P < 0.05) in amoeboid microglia in CCI (n = 9, 48 + 7) in comparison to sham (n = 6, 29 + 4) in the ipsilateral thalamus (Fig. 6E). There was a decrease in amoeboid microglia due to BMMNCs therapy (AUTO: n = 6, 33 + 2) in comparison to CCI, however that was not significant. BMMNCs therapy (n = 6, 43 + 8) resulted in an increase of ramified microglia in comparison to CCI (n = 9, 31 + 4) but it was not significant (Fig. 6D). The number of sham ramified microglia (n = 6, 29 + 4) in the thalamus were also similar to the CCI and not significant. We also compared the ratio of ramified vs amoeboid microglia, there was a significant increase (P < 0.03) in AUTO (n = 6, 1.3 + 0.3) vs CCI (n = 9, 0.7 + 0.1. Surprisingly, there was no difference between CCI (n = 9, 0.7 + 0.1) and sham (n = 6, 1 + 0.2, Fig. 6F).

## Discussion

PET ligand [^11^C]PBR-28 (PBR28suv) can be utilized to assess efficacy of BMMNCs therapy after TBI. There was a significant reduction in PBR28suv when compared to CCI alone (Fig. 2). BMMNCs therapy also improved spatial learning as measured by Morris Water Maze (Fig. 3). Ex vivo analysis of brain volume loss demonstrated that BMMNCs therapy preserved brain volume after TBI (Fig. 4). Additionally, BMMNCs therapy also resulted in a decrease in amoeboid-shaped microglia in the dentate gyrus of the hippocampus (Fig. 5) and a change in the ramified:amoeboid microglia in the thalamus (Fig. 6).

After TBI, due to the disruption of the blood brain barrier (BBB) there is an influx of macrophages and microglia are activated. Activated microglia can polarize towards a pro-inflammatory phenotype which can result in chronic neuroinflammation, oxidative stress and neuronal dysfunction. Activated microglia retract their processes and typically adopt an amoeboid morphology^6,11,12^. Morphological alterations may also be accompanied by alterations in mitochondria^13^. A protein that is up-regulated in expression after injury is the translocator protein (PBR), expressed by mitochondria in microglia, reactive astrocytes, blood-derived macrophages, endothelial and smooth muscle cells in the vasculature^18–20,33^. PBR can be detected in vivo in rodents and patients using positron emission topography (PET) ligands^1,26^. Evidence from human TBI patients using ligand [^11^C](R)PK11195 (PBR) suggests that amoeboid microglia/macrophages and/or reactive astrocytes remain present up to 17 years after injury^1^. We have demonstrated that there is a significant increase in [^11^C]PBR-28 standard uptake values (PBR28suv) after TBI in comparison to non-injured animals in a rodent model of TBI^26^. Recent evidence from Pannell et al.^34^ demonstrated that PBR is upregulated in astrocytes and microglia when stimulated with lipopolysaccharide (LPS), and this is reflected with an increase in PET imaging of PBR ligand [^18^F]DPA-713. In addition, they demonstrated PBR expression was significantly increased in microglia after AdTNF injections^34^. These data indicate that PBR is a good marker to target in order to observe increases of pro-inflammatory microglia in vivo.

Previously, we have demonstrated that BMMNCs therapy results in apoptosis of amoeboid-shaped microglia via cleaved caspase 3^15^. The attenuation of PBR28suv observed in the AUTO group (Fig. 2) is likely due to a reduction in amoeboid/activated microglia. Accompanying the decrease in PBR28suv, there was a significant decrease in the number of amoeboid/activated cells (IBA1 positive) in AUTO in comparison to CCI in the dentate gyrus (Fig. 5E). Previously, we had also observed a decrease in amoeboid-shaped microglia that co-stained with Cleaved Caspase 3 (CC3), a marker for apoptosis after BMMNCs therapy^15^. BMMNCs therapy decreases amoeboid microglia by causing them to go through apoptosis. In the current experiments, there was an increase in the ratio of ramified vs amoeboid cells in the thalamus (Fig. 6F). This indicates that BMMNCs therapy is reducing the pro-inflammatory environment created by amoeboid/activated microglia. Perhaps, the reduction in a pro-inflammatory environment due to BMMNCs therapy helps improve the latency to platform measurement in comparison to CCI alone (Fig. 3). We did not observe any significant differences in the probe trial in terms of the number of times hidden platform was crossed or the duration in 3× platform diameter. Previously, when we administered BMMNCs after CCI, we observed changes in the probe trial (circle and quadrant time)^15^. However, those experiments were done at 28 days after CCI and the current data (Fig. 3) is at 120–126 days after injury. Additionally, in the previous experiment, the rats were trained twice for the MWM prior to Probe testing, therefore the training paradigm was different than in the current study. Improvement in spatial learning is likely due to a reduction of amoeboid microglia and improved ratio of ramified vs amoeboid (Figs. 5 and 6). Additionally, BMMNCs therapy preserved brain volume in comparison to CCI, thereby preserving cellular structure. A limitation of the CT image analysis method is that it is not talking into account ventricular swelling (Fig. 4). Further techniques need to be developed in order to address this issue. Although the CT image analysis does not take into account ventricular swelling, we do observe better preserved hippocampal and cortical structures overall in the AUTO group. The preservation of hippocampal structures might account for the improvement in spatial learning (Fig. 3). Interestingly, brain volume loss correlates inversely with neurocognitive outcomes in patients^35^. Additional investigation needs to be done to better understand the relationship between changes in neurocognition and brain volume loss.

We demonstrate that PET ligand [^11^C]PBR-28 is a useful biomarker to indicate in vivo efficacy of BMMNCs treatment. Establishing a biomarker that indicates the changes due to cellular therapy is a valuable tool, as it can identify issues related to dosing and delineate responders from non-responders. [^11^C]PBR-28 will help advance the field in terms of determining outcome measures that allow for cell therapy for inflammatory-related trauma. The preclinical data also helps in setting the stage for the future development of BMMNCs treatment combined with PET-CT in patients with a traumatic brain injury.

## Data Availability

The datasets used and/or analyzed during the current study available from the corresponding author on reasonable request.
